# Quality of life in people living with HIV: a cross-sectional study in Ouagadougou, Burkina Faso

**DOI:** 10.1186/2193-1801-3-372

**Published:** 2014-07-21

**Authors:** Fidèle Bakiono, Laurent Ouédraogo, Mahamoudou Sanou, Sékou Samadoulougou, Patrice Wendpouiré Laurent Guiguemdé, Fati Kirakoya-Samadoulougou, Annie Robert

**Affiliations:** Pôle Epidémiologie et Biostatistique, Institut de Recherche Expérimentale et Clinique (IREC), Faculté de Santé Publique, Université catholique de Louvain, Clos Chapelle-aux-Champs 30, Brussels, 1200 Belgium; Unité de Formation et de Recherche en Sciences de la santé, Université de Ouagadougou, Ouagadougou 03, 03 BP 7021 Kragujevac, Burkina Faso; Institut Régional de Santé Publique de Ouidah, Ouidah, BP 384 Bénin

**Keywords:** Quality of life, HIV/AIDS, Burkina Faso, WHOQOL HIV-BREF

## Abstract

HIV/AIDS is a leading cause of death in most of sub-Saharan countries. HIV/AIDS impact on the quality of life of persons living with HIV in Burkina Faso hasn't been well documented. The aim of the study was to assess the quality of life in persons living with HIV and its associated factors.

A cross-sectional study was conducted in Ouagadougou. 424 persons living with HIV were included in the study according to their status with regard to Highly Active Anti Retroviral Treatment: 115 were not yet under treatment, 21 started the treatment within the three months preceding the enrolment and 288 were under treatment for at least 12 months. The quality of life was assessed through the WHOQOL HIV-BREF. Statistical comparisons were made using Mann Whitney U test, Kruskal-Wallis H test, Pearson's khi2 or Fisher's exact test. Correlations were appreciated using Spearman's rho. Logistic regression was used to examine associations between the quality of life scores and sociodemographic or clinical variables.

The mean global score of quality of life in all patients was 82.4. Better scores were recorded in the spiritual domain and worst scores in the environmental domain. Men had a higher global score than women (p < 0.001). Illiteracy was significantly associated with a lower quality of life (p = 0.001). Patients having support for medical treatment had a significantly better quality of life (p < 0.01). In multivariate analysis, being a man, having a support for medical care, getting older and self-perceived as healthy, were associated with a global score of quality of life higher than 77, that corresponds to the mid-range of the score in our data.

These findings suggest the importance of the socio-psychological support and of a good environment in order to improve the quality of life of people living with HIV, especially in women, in younger and in those having no support for medical care. In the environmental domain, actions of HIV services providers should focus on better accessibility to social and health care, promotion of income-generating activities especially for women and youth living with HIV.

## Background

In developing countries, especially in Western sub-Saharan Africa, leading causes of years of life lost are malaria, lower respiratory infections, diarrheal diseases and HIV/AIDS (Lopez et al. 2006). Since the introduction of Highly Active Antiretroviral Treatment (HAART) in the mid 1990's, treatments reduce the morbidity, the mortality related to HIV and HIV transmission (Hayward 2013). Longevity has been improved, but side effects of treatments are reported (Gakhar et al. 2013; Duran et al. 2001). The quality of life becomes an important area of concern for patients, health care providers, psychological and social support providers (Solomon et al. 2009). Studies conducted in sub Saharan Africa showed an improvement of the quality of life over time in people initiating treatment (Stangl et al. 2007; Jelsma et al. 2005). In Burkina Faso, where the prevalence in the general population is 1.1% (UNAIDS 2012), HIV remains a public health problem with large variations across subgroups(INSD/ICF International 2012; Kirakoya-Samadoulougou et al. 2013; Kirakoya-Samadoulougou et al. 2014). Efforts are made nationwide to improve the health and the wellbeing of people living with HIV (CNLS-IST/BF 2011). But there is few scientific evidence about their health related quality of life. One study conducted in Burkina Faso showed an evident increase in quality of life in people initiating the treatment over a 12 months follow-up time (Jaquet et al. 2013). But the quality of life of other people waiting for treatment or under treatment for more than 12 months remains insufficiently documented.

The aim of our study was to assess the quality of life in persons living with HIV and its associated factors.

## Methods

A cross-sectional study was conducted in Ouagadougou in September 2012. Recruitment took place in five structures that take care of People Living With HIV/AIDS (PLWHA) in the city. In their records, 22,788 persons were followed up for medical care and sociopsychological support. They represent 73.44% of the people followed up by all facilities in the Central Region, of which Ouagadougou is the capital city. Two of these facilities are integrated in the national health system and three are community-based. Those integrated in the national health system are the District Hospital of Boulmiougou (DHB) and the "Hôpital du Jour" a service of University Hospital Yalgado Ouédraogo (UHYO). The three community-based organizations are the Center for Information, Counseling and Documentation on AIDS and Tuberculosis (CICDoc), the Association African Solidarity (AAS), the Association Laafi la Viim (ALAVI). All those facilities offer anonymous voluntary counseling and testing (VCT) services, prevention counseling, medical treatment and monitoring, nutrition counseling, psychological and social support.

### Sampling

Setting the level of confidence at 95%, with a 5% precision with a maximal uncertainty, the required sample size was 385. This number was increased by 10% (39 people) leading to a total of 424 persons recruited. Determining the number of subjects to be recruited in each facility was made according to the size of the active list in each structure. Active list is defined as the number of PLWHA under treatment or not, and followed by the structure. Thus, in the district hospital of Boulmiougou which represents 35.38% of the total number of people followed by the 5 structures, 150 people were recruited. At the Hôpital du Jour (UHYO), representing 31.84% of this number, 135 persons were recruited. In the CICDoc, AAS and ALAVI representing respectively 15.10%, 9.20% and 8.49% of the active list of the five structures, 64, 39 and 36 persons were recruited, respectively. In each structure, to insure a sound representation of people living with HIV, recruitment took place from the routine consultation, and people were classified into three groups according to the status linked to the HAART: 1) HIV positive but not under HAART yet; 2) people who started HAART within the three months preceding the enrolment and 3) people under HAART for at least 1 year. The number in each group was determined in proportion to its size in the active list. Those who met criteria and who agreed to participate in the study were interviewed. We used trained interviewers, due to the high illiterate rate in the country and that is 43.7% in urban area of Burkina Faso (INSD 2008).

### Quality of life assessment tool

To assess the quality of life, we used the French version of WHOQOL HIV-BREF (Preau et al. 2007; Marcellin et al. 2007). For people who didn't understand French, Mooré version of WHOQOL HIV-BREF developed by Bakiono et al. was used. Mooré is the main language of communication in the country, whatever residence of individuals we consider (rural or urban) (INSD 2008). WHOQOL HIV-BREF is a 31-item tool which asks interviewee to rate his quality of life in many ways, during the last two weeks preceding the interview. WHOQOL HIV-BREF explores six domains of the quality of life : physical, psychological, level of independence, social relationships, environment and spirituality. Answers are rated on a five-point Likert scale from 1 to 5. Scoring was performed according to recommendations of the World Health Organization Quality of Life group (WHO 2002). Higher scores indicate better quality of life. In our sample, the Cronbach's alpha for reliability assessment was 0.85, indicating good internal consistency of the tool.

### Characteristics collected

Beside the data collected to assess the quality of life, socio demographic, economic and clinical data were collected by trained interviewers, fluent in French and in Mooré. Characteristics collected were: gender, age, marital status, occupation, level of education, knowledge attitudes and practices, risk behaviors, duration under treatment, time since being HIV-positive tested, serological status, great part in monthly expenses, disclosure of HIV status, experience in stigmatization.

### Statistical analysis

Analyses were performed using IBM SPSS 19.0. Groups were compared using Mann Whitney U test or Kruskal-Wallis H test for continuous variables and Pearson's χ ^2^ or Fisher's exact p-values for discrete or qualitative variables. Bivariate correlations were appreciated using the ρ of Spearman. For all analyses, a p-value lower than 0.05 was considered as significant. A cut off of global score of quality of life was set at 77, which represents the mid-range of the scale based on our data. A global score less than or equal to 77 was considered as bad and a global score higher than 77 as good. The robustness of the cut-off point was tested by comparing results using other cut-offs like 1) observed percentile 67 (upper tertile) which is data driven; 2) upper third of the scale which is not data driven and corresponds to 24 + 2(120–24)/3 = 88. Variables associated to good global score of quality of life in a bivariate analysis (p < 0.15) were included into a multiple logistic regression model.

### Ethical concerns

The present study has been approved by the Ethics Committee for Health Research from the Ministry of Health of Burkina Faso. All patients who agreed to participate in the study signed freely an informed consent.

## Results

Patients were 37.6 ± 8.5 years of age on average. 96.7% of them lived in Ouagadougou for an average time of 23 ± 14 years. The mean duration of HIV infection reported by interviewees was 5.7 ± 4.5 years and the mean time since initiation of HAART was 5.0 ± 3.1 years. Knowledge, attitudes and practices of interviewees have been assessed and showed that 5% of them didn't know transmission ways of HIV and 4.2% didn't know how to prevent from HIV. 69.5% of them didn't know the difference between AIDS and HIV positive. Among literate interviewees, the proportion of persons who didn't know the difference between AIDS and HIV positive was 60.4% while among illiterate interviewees, it was 88.3% (p < 0.001). Despite their status, 14.4% of the interviewees never used condom when having sex, 25.1% used it sometimes and 12.4% of them had no sex any longer. Among interviewees, 78.1% were asymptomatic, 18.5% were symptomatic and 3.4% were at AIDS stage. 35.6% didn't know how they contracted HIV while 57.3% reported heterosexual intercourse. 72.9% of respondents were taking Highly Active Antiretroviral Treatment (HAART). Characteristics of interviewees are shown in Table 1.

**Table 1:** **Characteristics of 424 persons living with HIV in Ouagadougou, Burkina Faso**

Interviewees characteristics	Proportion (%)	Mean ± SD Median [IQR]
Age (years)		37.6 ± 8.5
Gender		
Men	12.5	
Women	87.5	
Marital status		
Single	14.6	
Separated/divorced	19.6	
Widow	21.0	
Concubine	9.4	
Married	35.4	
Matrimonial system		
Monogamous	74.0	
Polygamous	26.0	
Profession		
Public or private sector employee	16.0	
Trader or informal sector	50.7	
Housewife	23.6	
Cultivator	3.1	
Student or pupil	2.8	
Jobless	3.8	
Religion		
Muslim	43.0	
Catholic	42.6	
Protestant	14.4	
Level of education		
Illiterate	32.8	
Primary school	31.8	
Secondary school	31.8	
University	3.6	
Time since HIV-positive tested (years)		5.7 ± 4.4
HIV transmission route reported		
Heterosexual intercourse	57.3	
Don't know	35.6	
Other	7.2	
Highly active antiretroviral treatment status		
No treatment	27.1	
Under treatment for less than 3 months	5.0	
Under treatment for at least 1 year	67.9	
HAART duration (years)		5 ± 3.1
Serology status		
Asymptomatic	78.1	
Symptomatic	18.5	
AIDS	3.4	
HIV services providers		
DHB	35.4	
HUYO	31.8	
CIC_Doc	15.1	
AAS	9.2	
ALAVI	8.5	
Household size		5 [3 ; 7]
Status disclosure with relative		
No	18.6	
Yes	81.4	
Sexual behavior risk		
No risk	60.5	
Risk	39.5	
Accepted by the family		
No	11.1	
Yes	88.9	
Greater part of monthly expenses		
Food	73.0	
Health	10.3	
Housing	7.9	
Other	8.8	

DHB = District Hospital of Boulmiougou; HUYO = University Hospital Yalgado Ouédraogo; CICDoc = Center for Information, Counseling and Documentation on AIDS and Tuberculosis; AAS = Association African Solidarity; ALAVI = Association Laafi la Viim.

The mean (SD) global score of quality of life in all patients was 82.4 (10.8). According to the domain, mean scores were 14.8(2.7) for physical domain; 13.6(2.7) for psychological domain; 13.3(2.5) for the level of independence domain; 13.4(2.9) for social relationships domain; 10.8(2.2) for environmental domain and 16.3(2.6) for religious and personal believes domain. In patients not under HAART, the global score of quality of life was 80.7(9.6). In patients under treatment for less than 3 months it was 80.2(14.2) and it was 80.2(10.9) for patients under treatment for at least 1 year ( *p = 0.07* ). Men had a global score of 87.2( 9.7) while women had a global score of 81.7(10.8) [*p < 0.001*]. Sharing or not one’s status with a family member was not significantly associated with better quality of life. Hospital or community-based nature of the structure of care had no significant impact on the quality of life of people living with HIV. Illiteracy was significantly associated with a lower quality of life (*p = 0.001*). Having support for medical treatment of the subject was significantly associated with better quality of life (*p < 0.01*). The perception of being ill was associated with poorer quality of life. Different domains scores and the overall score of quality of life related to socio-demographic and clinical characteristics are shown in Tables 2 and 3.

**Table 2:** **Quality of life domains scores by demographic characteristics of persons living with HIV in Ouagadougou, Burkina Faso (n = 424)**

Socio-demographic characteristics	Domain of quality of life						Global score of Quality Of Life (Mean ± SD)
	Physical domain (Mean ± SD)	Psychological domain (Mean ± SD)	Level of independence (Mean ± SD)	Social relationships (Mean ± SD)	Environmental domain (Mean ± SD)	Spiritual domain (Mean ± SD)	
Gender	*p = 0.14*	*p = 0.01*	*p = 0.008*	*p = 0.003*	*p = 0.001*	*p = 0.03*	*p < 0.001*
Men (n = 53)	15.4 ± 2.3	14.4 ± 2.7	14.1 ± 2.7	14.5 ± 2.5	11.7 ± 2.1	16.9 ± 2.5	87.2 ± 9.7
Women (n = 370)	14.7 ± 2.7	13.5 ± 2.6	13.2 ± 2.4	13.2 ± 2.9	10.7 ± 2.1	16.2 ± 2.6	81.7 ± 10.8
HIV services provider	*p = 0.22*	*p = 0.34*	*p = 0.003*	*p = 0.01*	*p < 0.001*	*p = 0.53*	*p = 0.05*
AAS (n = 39)	15.8 ± 2.4	14.4 ± 2.7	13.7 ± 2.2	13.3 ± 2.7	11.0 ± 1.8	16.3 ± 2.7	84.7 ± 10.3
DHB (n = 150)	14.9 ± 2.7	13.3 ± 2.6	13.7 ± 2.6	14.0 ± 2.7	11.4 ± 2.2	16.2 ± 2.7	83.7 ± 10.9
CIC-Doc (n = 64)	14.6 ± 2.7	13.5 ± 2.5	13.5 ± 2.6	13.3 ± 3.0	11.1 ± 1.9	15.9 ± 2.4	82.2 ± 11.1
ALAVI (n = 36)	14.7 ± 2.9	13.6 ± 2.7	13.0 ± 2.7	13.4 ± 3.1	9.9 ± 2.3	16.5 ± 3.0	81.2 ± 11.3
HUYO (n = 135)	14.7 ± 2.7	13.6 ± 2.8	12.7 ± 2.2	12.7 ± 2.9	10.2 ± 2.1	16.4 ± 2.4	80.6 ± 10.3
Level of education	*p = 0.02*	*p = 0.17*	*p < 0.001*	*p = 0.07*	*p = 0.31*	*p = 0.49*	*p = 0.007*
Illiterate (n = 139)	14.3 ± 2.6	13.3 ± 2.7	12.5 ± 2.3	12.9 ± 2.7	10.5 ± 2.0	16.2 ± 2.6	79.9 ± 10.1
Primary school (n = 135)	15.0 ± 2.6	13.7 ± 2.6	13.6 ± 2.1	13.7 ± 2.7	11.0 ± 2.2	16.4 ± 2.6	83.6 ± 10.4
Secondary school (n = 135)	15.1 ± 2.8	13.9 ± 2.7	13.7 ± 2.8	13.5 ± 3.1	10.9 ± 2.3	16.3 ± 2.5	83.6 ± 11.6
University (n = 15)	15.6 ± 1.8	12.9 ± 2.6	13.9 ± 2.2	13.7 ± 3.1	11.0 ± 2.4	15.1 ± 3.2	82.3 ± 10.3
Marital status	*p = 0.49*	*p = 0.13*	*p = 0.55*	*p = 0.01*	*p = 0.73*	*p = 0.87*	*p = 0.53*
Single (n = 62)	14.4 ± 3.1	13.1 ± 3.1	13.4 ± 2.8	13.2 ± 3.4	10.6 ± 2.7	16.0 ± 3.0	80.9 ± 13.2
Married (n = 150)	15.0 ± 2.5	13.9 ± 2.3	13.5 ± 2.1	13.8 ± 2.7	10.9 ± 2.1	16.3 ± 2.3	83.7 ± 9.8
Partner (n = 40)	15.0 ± 2.4	13.1 ± 3.4	13.2 ± 2.6	13.6 ± 3.0	10.5 ± 2.2	16.1 ± 2.5	81.6 ± 11.6
Widower (n = 89)	14.5 ± 2.8	14.0 ± 2.3	13.1 ± 2.3	13.0 ± 2.4	10.8 ± 2.0	16.4 ± 2.7	82.0 ± 9.7
Divorced/Separated (n = 83)	15.1 ± 2.7	13.1 ± 2.7	13.1 ± 2.9	12.9 ± 3.0	10.9 ± 2.0	16.3 ± 2.5	81.7 ± 11.1
Matrimonial system	*p = 0.17*	*p = 0.15*	*p = 0.16*	*p = 0.27*	*p = 0.07*	*p = 0.83*	*p = 0.16*
Monogamous (n = 99)	15.1 ± 2.3	14.0 ± 2.4	13.2 ± 2.2	13.9 ± 2.4	11.0 ± 2.0	16.4 ± 2.2	83.9 ± 9.2
Polygamous (n = 37)	14.4 ± 2.8	13.5 ± 2.2	13.6 ± 1.6	13.4 ± 2.9	10.4 ± 2.1	16.1 ± 2.8	81.6 ± 10.1
Religion	*p = 0.96*	*p = 0.39*	*p = 0.35*	*p = 0.21*	*p = 0.08*	*p = 0.006*	*p = 0.78*
Muslims (n = 182)	14.9 ± 2.7	13.6 ± 2.6	13.3 ± 2.3	13.6 ± 2.8	10.9 ± 2.2	16.1 ± 2.4	82.7 ± 10.5
Catholics (n = 180)	14.8 ± 2.6	13.4 ± 2.6	13.2 ± 2.7	13.2 ± 2.7	10.9 ± 2.0	16.1 ± 2.7	81.9 ± 10.6
Protestants (n = 61)	14.8 ± 2.8	13.9 ± 3.0	13.7 ± 2.2	13.1 ± 3.5	10.3 ± 2.4	17.1 ± 2.6	83.2 ± 10.7
Profession	*p = 0.10*	*p < 0.005*	*p = 0.001*	*p = 0.03*	*p = 0.30*	*p = 0.16*	*p = 0.007*
Public, private employees (n = 68)	15.6 ± 2.3	14.3 ± 2.2	14.5 ± 2.2	13.7 ± 3.0	11.3 ± 2.2	16.6 ± 2.6	86.3 ± 9.5
Trade & Informal sector (n = 215)	14.8 ± 2.7	13.6 ± 2.5	13.2 ± 2.4	13.2 ± 2.8	10.7 ± 2.1	16.2 ± 2.6	82.0 ± 10.2
Housewives (n = 100)	14.6 ± 2.5	13.3 ± 2.6	13.1 ± 2.2	13.7 ± 2.7	10.8 ± 2.2	16.4 ± 2.4	82.0 ± 10.5
Farmers (n = 13)	14.3 ± 3.3	14.9 ± 2.2	12.7 ± 2.3	14.4 ± 2.1	10.9 ± 2.6	17.0 ± 2.4	84.6 ± 11.7
Student & pupil (n = 12)	13.9 ± 3.4	10.9 ± 3.7	12.3 ± 3.6	13.1 ± 3.6	10.1 ± 2.5	14.3 ± 3.4	74.8 ± 16.5
Jobless (n = 16)	14.0 ± 3.7	12.1 ± 4.1	12.4 ± 4.1	11.3 ± 3.3	10.9 ± 1.7	15.8 ± 2.6	76.7 ± 14.2
Family cohesion	*p = 0.03*	*p = 0.02*	*p = 0.40*	*p = 0.02*	*p = 0.005*	*p = 0.001*	*p = 0.001*
Hassle (n = 75)	14.3 ± 2.9	12.9 ± 2.7	13.1 ± 2.6	12.7 ± 3.2	10.2 ± 2.4	15.4 ± 2.7	78.7 ± 11.2
Without hassle (n = 343)	15.0 ± 2.6	13.7 ± 2.6	13.3 ± 2.5	13.5 ± 2.8	11.0 ± 2.1	16.5 ± 2.5	83.3 ± 10.5

DHB = District Hospital of Boulmiougou; HUYO = University Hospital Yalgado Ouédraogo; CICDoc = Center for Information, Counseling and Documentation on AIDS and Tuberculosis; AAS = Association African Solidarity; ALAVI = Association Laafi la Viim.

**Table 3:** Quality of life domains scores by clinical characteristics of persons living with HIV in Ouagadougou, Burkina Faso (n = 424*)

Clinical characteristics	Domain of quality of life						Global score of quality of life (Mean ± SD)
	Physical domain (Mean ± SD)	Psychological domain (Mean ± SD)	Level of independence (Mean ± SD)	Social relationships (Mean ± SD)	Environmental domain (Mean ± SD)	Spiritual domain (Mean ± SD)	
Serology status	*p = 0.003*	*p = 0.52*	*p = 0.001*	*p = 0.32*	*p = 0.40*	*p = 0.15*	*p = 0.02*
Asymptomatic (n = 325)	15.1 ± 2.5	13.7 ± 2.5	13.5 ± 2.4	13.5 ± 2.8	10.9 ± 2.2	16.4 ± 2.5	83.4 ± 10.3
Symptomatic (n = 77)	14.0 ± 2.7	13.3 ± 3.1	12.4 ± 2.7	13.0 ± 2.8	10.6 ± 2.2	16.2 ± 2.4	79.7 ± 11.5
AIDS (n = 14)	14.2 ± 3.1	13.0 ± 2.8	14.2 ± 2.8	13.5 ± 3.4	10.7 ± 2.1	14.7 ± 3.4	80.5 ± 12.1
Highly Active Antiretroviral Treatment status	*p = 0.002*	*P < 0.001*	*p = 0.92*	*p = 0.44*	*p = 0.87*	*p = 0.78*	*p = 0.08*
No treatment (n = 110)	14.2 ± 2.7	12.8 ± 2.6	13.2 ± 2.4	13.6 ± 2.4	10.9 ± 2.1	16.3 ± 2.3	82.8 ± 11.1
Under treatment (n = 312)	15.1 ± 2.6	13.9 ± 2.6	13.3 ± 2.5	13.3 ± 3.0	10.8 ± 2.2	16.2 ± 2.7	81.1 ± 9.7
Perceived health state	*p < 0.001*	*p < 0.001*	*p < 0.001*	*p < 0.001*	*p = 0.001*	*p = 0.005*	*p < 0.001*
Good health state (n = 270)	15.5 ± 2.4	14.1 ± 2.4	13.8 ± 2.2	13.8 ± 2.8	11.1 ± 2.2	16.6 ± 2.4	85.2 ± 9.7
Median health state (n = 131)	13.8 ± 2.7	12.9 ± 2.7	12.8 ± 2.5	12.8 ± 2.7	10.4 ± 1.9	15.8 ± 2.8	78.8 ± 10.5
Bad health state (n = 23)	12.0 ± 1.8	10.9 ± 2.8	10.2 ± 3.0	11.5 ± 3.2	9.8 ± 1.9	15.2 ± 2.7	69.9 ± 10.6
Perceived disease	*p < 0.001*	*p < 0.001*	*p < 0.001*	*p = 0.007*	*p = 0.01*	*p = 0.01*	*p < 0.001*
Yes (n = 142)	13.7 ± 2.8	12.6 ± 2.9	12.3 ± 2.6	12.8 ± 2.9	10.5 ± 2.2	15.8 ± 2.7	77.9 ± 11.3
No (n = 244)	15.5 ± 2.4	14.3 ± 2.4	13.8 ± 2.2	13.7 ± 2.8	11.0 ± 2.1	16.5 ± 2.5	85.1 ± 9.6
Sexual behavior	*p = 0.04*	*p = 0.32*	*p = 0.82*	*p = 0.93*	*p = 0.12*	*p = 0.52*	*p = 0.35*
Risk (n = 165)	14.5 ± 2.6	13.5 ± 2.5	13.3 ± 2.3	13.3 ± 3.0	10.6 ± 2.3	16.4 ± 2.3	81.9 ± 10.6
No risk (n = 253)	15.0 ± 2.7	13.6 ± 2.8	13.3 ± 2.6	13.4 ± 2.8	11.0 ± 2.1	16.1 ± 2.8	82.7 ± 11.0
Support for medical care	*p = 0.16*	*p = 0.28*	*p = 0.55*	*p < 0.001*	*p = 0.57*	*p = 0.04*	*p = 0.02*
Yes (n = 266)	15.0 ± 2.7	13.7 ± 2.6	13.4 ± 2.3	13.8 ± 2.7	10.9 ± 2.1	16.5 ± 2.5	83.4 ± 10.4
No (n = 157)	14.6 ± 2.7	13.4 ± 2.8	13.1 ± 2.7	12.6 ± 3.1	10.7 ± 2.2	15.9 ± 2.7	80.5 ± 11.2

*Total may differ according to characteristics.

In our sample, overall general health perception was significantly and positively correlated with all domains of the quality of life, as for global score of quality of life (*p < 0.01*).

In multivariate analysis, being a man, having a support for medical care, older age and self-perceived as healthy, were associated with a global score of quality of life higher than 77 (Table 4). Being a woman, self perceived as ill result with lower score of quality of life (Table 5).

**Table 4:** **Factors associated with a higher value of global score of the quality of life in persons living with HIV in Ouagadougou, Burkina Faso**

	Total n*	Global score of QOL >77 n (%)	Univariate Unadjusted OR (95% CI)	Multivariate Adjusted OR (95% CI)	P for Adjusted OR
Conjugal life					
Alone	234	160 (68.4)	1		
In couple	190	146 (76.8)	1.53 (0.99-2.37)		
Gender					
Man	53	45 (84.9)	1	1	
Woman	370	260 (70.3)	0.42 (0.19 - 0.92)	0.37 (0.15 - 0.88)	0.02
Support for medical care					
No	157	104 (66.2)	1	1	
Yes	266	201 (75.6)	1.57 (1.02 - 2.43)	2.18 (1.32 - 3.61)	0.002
Age (year)					
19 - 24	26	12 (46.2)	1	1	0.01
25 - 34	135	90 (66.7)	2.33 (0.99 - 5.5)	2.68 (1.04 -6.89)	0.04
35 - 44	170	129 (75.9)	3.67 (1.57 - 8.56)	4.04 (1.55 - 10.51)	0.004
45 - 65	91	73 (80.2)	4.73 (1.87 - 11.96)	4.31 (1.52 - 12.22)	0.006
Nature of care facility					
Hospital	285	200 (70.2)	1		
Community based	139	106 (76.3)	1.35 (0.85 - 2.17)		
Health expense as greater part of monthly spending					
No	72	45 (62.5)	1		
Yes	352	261 (74.1)	1.72 (1.00 - 2.93)		
Highly Active Antiretroviral Treatment					
No	110	72 (65.5)	1		
Yes	312	233 (74.7)	1.55 (0.97 - 2.48)		
School					
No	139	93 (66.9)	1		
Yes	285	213 (74.7)	1.46		
Self perceived as ill					
No	244	197 (80.7)	1	1	
Yes	142	79 (55.6)	0.29 (0.18 - 0.47)	0.31 (0.19 - 0.50)	<0.001

*Total number may differ according to factor.

**Table 5:** **Multivariate logistic regression model for predicting chances of a global score of quality of life higher than 77 in persons living with HIV in Ouagadougou, Burkina Faso**

Variables	β ± SE	P-value
Living in couple (yes = 1; no = 0)	0.32 ± 0.27	0.23
Being a woman (yes = 1; no = 0)	−0.80 ± 0.46	0.08
Having support for medical care (yes = 1; no = 0)	0.65 ± 0.27	0.01
Age (years)		
25 - 34 (yes = 1; no = 0)	0.90 ± 0.49	0.06
35 - 44 (yes = 1; no = 0)	1.30 ± 0.50	0.01
45 - 65 (yes = 1; no = 0)	1.42 ± 0.55	0.01
Hospital nature of care facility (yes = 1; no = 0)	0.05 ± 0.27	0.85
Health expense as greater part of monthly spending (yes = 1; no = 0)	0.25 ± 0.30	0.41
Under highly active antiretroviral treatment (yes = 1; no = 0)	0.16 ± 0.28	0.56
Having been to school (yes = 1; no = 0)	0.26 ± 0.26	0.32
Self perceived as ill (yes = 1; no = 0)	−1.12 ± 0.25	<0.001
Constant	−0.02 ± 0.77	0.97

When using a slightly cut-off point of 88, that corresponds to the upper third of the scale, associations were comparable (Gender effect: OR = 0.37 changed to OR = 0.43, self perceived as ill effect: OR = 0.31 changed to OR = 0.43). Associations also maintained when reducing the cut-off point.

## Discussion

Our study involved 424 persons living with HIV. A majority of them were small traders, employees in informal sector and housewives. According to national data in urban area, like Ouagadougou, the population is mainly working in services and in trading (INSD 2008), which is consistent with our findings. The most common route of transmission reported by interviewees was heterosexual intercourse which is consistent with data from sub-Saharan Africa and specifically general data of Burkina Faso, reporting heterosexual intercourse as the main route of transmission (UNAIDS 2012).

A majority of our patients declared to be asymptomatic: 76.6%. It was almost the same proportion which was under treatment. Free access to treatment could have enhanced the proportion of persons under treatment leading to a better health status, without symptoms.

Our study showed that 14.4% of respondents never used condoms when having sex and 25.1% of them used it sometimes. These proportion of sexual risk behavior contrasts with the proportion of respondents who knew the routes of transmission of HIV, which was 95%. These sexual risk behaviors were highlighted by Guira et al. in their study on sexuality and the risk of sexual transmission (Guira et al. 2013). In their study involving 87 heterosexual serodiscordant couples, it was found that while 97.5% of couples were aware of the use of condoms as a mean of preventing the HIV transmission, nearly 60% of them did not use a condom systematically. The privacy concerns and the desire to give birth (for women) were the main reasons for the inconsistent use of condoms.

In our sample, the global score of quality of life was 82.4 ± 10.8. It is a lower mean score than what has been found in Ethiopia by Deribew et al. with a mean score set at 91.9 in HIV-positive people (Deribew et al. 2009; Deribew et al. 2013). In these studies mean score was 80.3 in co-infected HIV-TB people, close to our score. In Vietnam, Tran reported 76.2, a slightly lower mean score of overall quality of life than our mean value, but close to the cut-off value we used in the present study (Tran et al. 2012).

Whatever domain of quality of life we consider, scores were higher among men compared to women. These higher scores recorded in men may be explained by the socioeconomic level of men, generally higher than that of women, facilitating a better dealing with the disease. However, our results regarding quality of life and gender suggest more investigations. In rural Uganda, Stangl et al. didn't find any significant difference in the quality of life according to gender (Stangl et al. 2012). In Nigeria, no difference has been found in quality of life scores in regard with gender (Ogbuji and Oke 2010). In Burkina Faso, Jaquet et al. found a slight difference with advantage for men in mental health summary score and advantage for women in physical health summary score (Jaquet et al. 2013). In a large literature review on quality of life across countries and continents, Chandra et al. showed that in most of them, women reported lower quality of life than men (Chandra et al. 2009). These results, which included patients at different stages of the disease and under treatment, are consistent with our findings.

In our findings, the highest scores were recorded in the spiritual domain and the lowest scores were found in the environmental domain. In Burkina Faso, 99.6% of the population practice a religion (INSD 2008). As religions recommend the seek of heaven's wellbeing, instead of earthy happiness, religion practices of our subjects and the distress situation in which they are living due to the disease could explain the high scores recorded in the spiritual domain. This demonstrates how people in distress refer to religion to better accept their situation. In a study conducted in Ethiopia (Deribew et al. 2013), better scores were recorded in spiritual domain and the worst in environmental scores, results consistent with ours. The lower scores in environmental domain could be explained by the economical situation of our respondents dominated by small traders, employees of informal sector and housewives. As environmental domain explores, among others, home environment, financial resources, health and social care accessibility and quality, the lower economic level in our sample can explain lower scores in that domain.

Our study showed that in the HIV/AIDS care clinics, people living with HIV were predominantly under treatment. But they were still 27% not yet under treatment. Starting treatment for people living with HIV is based on the level of CD4 and viral load (CNLS-IST/BF 2008). However, in Burkina Faso, starting treatment may be delayed for financial reasons. In January 2010 the country started with free treatment for people in need; a rapid growth in the number of people on treatment has subsequently been observed. Prior to 2010, a significant decrease in the price of Highly Active Antiretroviral Treatments has been conceded in 2005 followed by another decrease in 2008 (Kouanda et al. 2010). In our study, patients who started treatment before 2005 represented 20% of the number of people under treatment. The large majority was under treatment since 2005. These results suggest that the high cost of treatment may be the reason of the small proportion of people on treatment by 2005, explaining at the same time the average duration of the initiation of treatment towards 5 years.

Our results didn't show significantly higher scores according to the duration under treatment except for spirituality domain where being under treatment since 2005 showed better scores than before 2005. Handajani et al. in their study involving 88 outpatients living with HIV showed a significant association between duration of HAART and physical domain (Handajani et al. 2012). In Uganda, Bajunirwe et al. showed that duration of less than one year was significantly associated with a lower physical health summary (Bajunirwe et al. 2009).

Our findings highlighted that being a man, having a support for medical care, ageing and self-perceived as healthy were associated with a global score of quality of life higher than 77. In a previous study in Burkina Faso, authors found that older age (> = 35 years) et gender (be a man) were associated with better mental health summary score (Jaquet et al. 2013). Our results are also consistent with the study of Skevington et al. with regard to age (Skevington 2012). No matter the domain of quality of life, scores they found were better in older people. Adewuya et al. also found in Nigeria that lower education level and poor social support were associated with poor quality of life (Adewuya et al. 2008).

## Limitations

The whole country has about 100 structures for taking care of people living with HIV. Our study was conducted in Ouagadougou, the capital city of Burkina Faso, in five HIV services providers and during a one-month enrolment period. Results may not reflect nationwide overview, especially in rural areas. However, these five structures were chosen for their large queues of people followed up and they are a good representation of people living with HIV in Ouagadougou or in urban areas of Burkina Faso.

## Conclusion

To our knowledge, it is the first time quality of life in persons living with HIV has been assessed using WHOQOL HIV, without restricting to people initiating anti retroviral treatment in Burkina Faso. Our findings showed worst scores in the environmental domain, with better scores of quality of life associated with good family cohesion, with a support for medical care, with a self-perceived as healthy, with ageing and in man. These findings highlight the importance of the socio-psychological support and of a good environment in order to improve the quality of life of people living with HIV, especially in women, at a younger age and in those having no support for medical care. In the environmental domain, actions of HIV services providers should focus on a better accessibility to social and health care, on the promotion of income-generating activities, especially for women and youth living with HIV.

## Abbreviations

AAS: Association African Solidarity
ALAVI: Association Laafi la Viim
CICDoc: Center for Information, Counseling and Documentation on AIDS and Tuberculosis
DHB: District Hospital of Boulmiougou
HAART: Highly Active Antiretroviral Treatment
HIV/AIDS: Human Immunodeficiency Virus/Acquired Immunodeficiency Syndrome
PLWHA: People Living With HIV/AIDS
UHYO: University Hospital Yalgado Ouédraogo
VCT: Voluntary Counseling and Testing
WHO: World Health Organization
WHOQOL: World Health Organization Quality of Life
WHOQOL HIV-BREF: World Health Organization Quality of Life assessment brief tool in patients with Human Immunodeficiency Virus infection.
